# Virulence Factor Genes and Antimicrobial Susceptibility of *Staphylococcus aureus* Strains Isolated from Blood and Chronic Wounds

**DOI:** 10.3390/toxins13070491

**Published:** 2021-07-14

**Authors:** Anna Budzyńska, Krzysztof Skowron, Agnieszka Kaczmarek, Magdalena Wietlicka-Piszcz, Eugenia Gospodarek-Komkowska

**Affiliations:** 1 Department of Microbiology, Ludwik Rydygier Collegium Medicum in Bydgoszcz, Nicolaus Copernicus University in Toruń, 9 M. Skłodowska-Curie St., 85-094 Bydgoszcz, Poland; a.budzynska@cm.umk.pl (A.B.); a.kaczmarek@cm.umk.pl (A.K.); gospodareke@cm.umk.pl (E.G.-K.); 2 Department of Theoretical Foundations of Biomedical Sciences and Medical Computer Science, L. Rydygier Collegium Medicum in Bydgoszcz, Nicolaus Copernicus University in Toruń, 9 M. Skłodowska-Curie St., 85-094 Bydgoszcz, Poland; mpiszcz@cm.umk.pl

**Keywords:** bacteremia, chronic wound, resistance, *Staphylococcus aureus*, virulence genes

## Abstract

*Staphylococcus aureus* is one of the predominant bacteria isolated from skin and soft tissue infections and a common cause of bloodstream infections. The aim of this study was to compare the rate of resistance to various antimicrobial agents and virulence patterns in a total of 200 *S. aureus* strains isolated from patients with bacteremia and chronic wounds. Disk diffusion assay and in the case of vancomycin and teicoplanin-microdilution assay, were performed to study the antimicrobial susceptibility of the isolates. The prevalence of genes encoding six enterotoxins, two exfoliative toxins, the Panton–Valentine leukocidin and the toxic shock syndrome toxin was determined by PCR. Of the 100 blood strains tested, the highest percentage (85.0%, 31.0%, and 29.0%) were resistant to benzylpenicillin, erythromycin and clindamycin, respectively. Out of the 100 chronic wound strains, the highest percentage (86.0%, 32.0%, 31.0%, 31.0%, 30.0%, and 29.0%) were confirmed as resistant to benzylpenicillin, tobramycin, amikacin, norfloxacin, erythromycin, and clindamycin, respectively. A significantly higher prevalence of resistance to amikacin, gentamicin, and tobramycin was noted in strains obtained from chronic wounds. Moreover, a significant difference in the distribution of *sea* and *sei* genes was found. These genes were detected in 6.0%, 46.0% of blood strains and in 19.0%, and 61.0% of wound strains, respectively. Our results suggest that *S. aureus* strains obtained from chronic wounds seem to be more often resistant to antibiotics and harbor more virulence genes compared to strains isolated from blood.

## 1. Introduction

*Staphylococcus aureus* is a common opportunistic pathogen that causes a variety of infections due to the presence of many colonization factors and virulence factors. It is one of the most frequent causes of skin and soft tissue infections (SSTIs) such as skin abscesses, furuncles, impetigo, and wound infections. Some of them, especially in patients with risk factors (diabetics, patients during immunosuppressive or cancer therapy, patients with indwelling catheters, HIV/AIDS), may progress to severe infections and require hospitalization. *S. aureus* is also a leading cause of serious infections, such as bacteremia (*S. aureus* bacteremia, SAB) or infective endocarditis, which can have serious consequences for the patient. High morbidity and mortality are associated especially with the widespread occurrence of methicillin-resistant *S. aureus* (MRSA) strains. Resistance to all β-lactams (except for the latest generation of cephalosporins) and other antibiotics commonly used limits therapeutic options for treating staphylococcal infections [1,2,3].

Among virulence factors produced by this microorganism, the pore-forming toxins (e.g., hemolysins, leukotoxins), enzymes (e.g., nucleases, staphylokinase, coagulases), epidermolytic toxins, and superantigens (SAgs) can be distinguished.

Panton–Valentine leukocidin (PVL)—encoded by two genes, *luk-S-PV*, and *luk-F-PV*—is an extracellular protein with dermonecrotic and leucocidal functions. It has cytotoxic activity against mammalian neutrophils, monocytes, and macrophages. The toxin can be produced both by community-acquired MRSA (CA-MRSA) as well as community-acquired methicillin-susceptible *S. aureus* (CA-MSSA) strains. PVL-positive strains usually cause SSTIs, but they are also associated with severe infections, including septic arthritis, bacteremia, necrotizing pneumonia, and purpura fulminans [4,5].

Exfoliative toxins (ETs) are specific serine proteases that recognize and hydrolyze desmosome proteins in the skin and, therefore, play a role in host colonization and the invasion of injured mucosa and skin. ETs are causative agents for localized epidermal infections and generalized disease—staphylococcal scalded skin syndrome (SSSS). Serotypes ETA, ETB, and ETD are also associated with septic shock and may increase the severity of the infection [6,7].

Toxic shock syndrome toxin-1 (TSST-1) and staphylococcal enterotoxins (SEs), with their superantigenic properties, may contribute to the development of serious infections due to the activation of enormous numbers of T lymphocytes. TSST-1 is a bacterial exotoxin produced by approximately 5–25% of *S. aureus* strains isolated from samples of different origins [8,9]. It is responsible for menstrual and non-menstrual cases of toxic shock syndrome, a severe and potentially fatal disease in humans or for neonatal toxic shock syndrome-like exanthematous disease. SEs are subdivided into classical SEs with emetic activity and SE-like proteins with no or relatively low emetic activity [10]. SEs play an important role in staphylococcal food poisoning, but these virulence factors are associated also with the pathogenesis of several other human diseases, including pneumonia, sepsis-related infections, or toxic shock syndrome. Futhermore, SEB is capable of inhibiting keratinocyte proliferation and migration and may delay wound closure [11,12].

Recent studies have shown that staphylococcal SAgs affect the site of infection to cause tissue pathology and are crucial in the development and progression of sepsis and infective endocarditis [13]. SAgs produced by *S. aureus* colonizing the wounds may also play a major inhibitory role in the healing of chronic wounds because of the recurrent exposure to those virulence factors [14].

This study aimed to compare drug susceptibility and virulence patterns in *S. aureus* strains isolated from patients with SAB or a chronic wound.

## 2. Results

### 2.1. Antibiotic Susceptibility Analysis

All the strains analysed were susceptible to ceftaroline, vancomycin, teicoplanin, tigecycline, linezolid, and rifampicin. The MIC range for the vancomycin was 0.5–1.5 µg·mL^−1^ and for teicoplanin 0.5–1.0 µg·mL^−1^. The resistance to antibiotics was observed more frequently in strains isolated from wounds than in strains isolated from blood (276 (18.4%) versus 222 (14.7%)) (*p* = 0.007). In the two groups, the highest resistance was observed to benzylpenicillin (Table 1). Moreover, in the group of wound strains, high percentages of strains not susceptible to tobramycin (32.0%), norfloxacin (31.0%), amikacin (31.0%), erythromycin (30.0%), and clindamycin (29.0%) were determined, whereas among blood strains, 31.0% and 29.0% of strains were resistant to erythromycin and clindamycin, respectively. Only with aminoglycosides were there statistically significant differences; higher prevalence of resistance to these antibiotics was observed in strains isolated from chronic wounds (*p* < 0.05). Of the 200 S. aureus strains, 23 (23.0%) blood strains and 21 (21.0%) wound strains were identified as MRSA. An iMLS_B_ and cMLS_B_ (constitutive MLS_B_ resistance) phenotype was observed in 19 (19.0%) and 11 (11.0%) of the blood strains, respectively, and in 14 (14.0%) and 14 (14.0%) of the wound strains, respectively. In the present study, 34 (34.0%) and 33 (33.0%) strains isolated from blood and chronic wounds were multidrug-resistant, of which 23 strains were methicillin-susceptible (Table 2). Only 6 (6.0%) wound strains and 14 (14.0%) blood strains were susceptible to all antibiotics tested. Resistance to one antimicrobial agent was seen in the majority of blood and wound strains (46.0% and 37.0%, respectively). The most frequent resistance phenotypes besides the P (40.5% of all strains) were P-E-CC (10.3% of MSSA strains) and P- NOR-AMK-TOB-E-CC (34.1% of MRSA strains).

### 2.2. Frequency of Virulence Genes

The frequencies of virulence genes are listed in Table 3. The most prevalent toxin-encoding genes detected, regardless of the origin of the S. aureus strains, were sei and seg. About one-fifth of the wound strains had sed or eta genes (20.0% and 19.0%, respectively). The etb gene was present only in one blood strain. Compared with blood strains, the carriage rates for sea and sei genes in wound strains were significantly higher (*p* < 0.05).

In the present study, 106 (67.9%) of the 144 MSSA strains and 40 (90.9%) of the 44 MRSA strains harbored one or more virulence genes. The genes that encode SEI and SEG were also the most common genes both in MSSA and MRSA strains (Table 3). Furthermore, the virulence gene detected among a large number of MRSA strains included *sed* (40.9%), whereas *etb* was not detected among those strains. Twenty-five (16.0%) and twenty-three (14.7%) MSSA strains harbored *eta* and *sea* gene, respectively, compared with two of the 44 MRSA strains. The statistically significant differences between MRSA and MSSA were found in the presence of *sed*, *seg*, *sei* and *eta* genes (*p* < 0.05).

In the tested populations, 39 virulence genes profiles were observed, including 12 common for both groups, 17 typical only for wound strains, and 10 for blood strains. None of the strains simultaneously harbored all tested genes. Twenty-three (57.5%) wound strains carried at least three virulence genes, whereas among blood strains this ratio was significantly lower (22.0%). Among 100 *S. aureus* strains obtained from a chronic wound, the most were found to be positive for two (25.0% of strains) and for three (25.0% of strains) virulence genes. In contrast, of all blood strains, 37.5% did not carry any of the tested genes. The most frequent combination detected among both groups was *seg-sei* (15.0% of the wound strains and 21.0% of the blood strains). All but 22 (78.0%) of the wound strains and 57 (57.0%) of the blood strains harbored at least one enterotoxin gene. Different patterns of the presence of toxin encoding genes in wound and blood strains are presented in Table 4.

### 2.3. Relationship between the Resistance to Antibiotics and Presence of Toxin Genes

To select independent factors associated with the detected resistance to antibiotics, the GLMM has been fitted to the data. The results of the uni- and multivariable models’ estimation are shown in Table 5. The results of the univariable analysis indicate the possible association between the antimicrobial resistance and the strain origin, as well as the presence of the sed, seg, and sei gene. However, in the multivariable analysis, the presence of the sed and seg gene appeared as the only covariate significantly associated with the antimicrobial resistance. The performed analysis shows that the presence of the sed gene in the examined sample increases the odds of the resistance to antibiotics by over nine times on average.

## 3. Discussion

*S. aureus* invades different body sites and causes a wide range of infections from mild skin and soft tissue infections to life-threatening diseases. Bloodstream infections remain especially associated with significant mortality, despite the improvements in the treatment of staphylococcal infections [15]. SAB may be associated with infective endocarditis, osteomyelitis, and other metastatic infections. Chronic wounds, often caused by this pathogen, can also be a source of bloodstream infections. Furthermore, non-healing wounds due to bacterial infections and biofilm formation are related to increased health costs [16].

A significant concern for public health are MRSA strains. Although, the decline in the resistance percentage was noted in recent years in some countries (e.g., the United Kingdom, Ireland, Germany, Portugal), MRSA remains an important pathogen involved in community- and health care-associated infections [17]. The frequency of isolation of MRSA strains from chronic wounds is varied and depends not only on the country, but also on the population of wound patients studied. Risk factors for infection with a multi-drug resistant microorganism include previous hospitalization or stay in a chronic care center (the possibility of cross-contamination of patients’ wounds or through the hands of medical staff), or previous antibiotic therapy. Moreover, chronic wounds are a good environment for the development of many different bacteria, which promotes horizontal gene transfer and selection of resistant strains [18,19]. MRSA bloodstream infection, in turn, is associated with high mortality, especially in intensive care patients. In the presented study, 21.0% of *S. aureus* isolated from chronic wounds were found to be MRSA, whereas Shettigar et al. [20] identified in diabetic foot ulcer patients an almost three times higher percentage of methicillin-resistant strains. The incidence of methicillin resistance that we have noted among strains isolated from blood was similar (23.0%) to that of wound strains. A statistically significant difference was indicated between the two groups of strains in resistance to amikacin (12.0% vs. 31.0%), gentamicin (1.0% vs. 13.0%) and tobramycin (14.0% vs. 32.0%). Moreover, among the strains isolated from blood, there was a lower percentage of strains resistant to norfloxacin, but this difference was not statistically significant. However, a similar percentage of blood (11.0%) and wound (14.0%) strains had a constitutive mechanism of MLS_B_ resistance. Compared to it and compared to strains isolated from blood, the inducible MLS_B_ mechanism was slightly more frequently reported in strains derived from chronic wounds (19.0 vs. 14.0%).

Benzylpenicillin resistance was found in most strains in the present study and the studies by other authors [20,21,22,23,24], regardless of the clinical samples from which they were recovered. Among the remaining antibiotics, the highest percentage of all strains tested showed resistance to clindamycin (30.0%), erythromycin (29.5%), and norfloxacin (27.0%). Similar findings (19.8%, 28.7%, and 27.7%) were reported by Pomorska-Wesołowska et al. [25] in Southern Poland on strains isolated from SAB and pneumonia. Other studies conducted by Wang et al. [22], Yu et al. [21], and Liang et al. [26] indicated that the frequencies of erythromycin and clindamycin resistance among isolates collected from blood were significantly higher and amounted to 65.0%, 61.8% and 46.7% for the former and 58.3%, 48.3% and 40.0% for the latter of these antibiotics. The widespread use of some macrolides against infections caused by various bacteria promotes among staphylococci colonizing the human body the selection of strains resistant to these antibiotics. In contrast, clindamycin is an alternative to beta-lactam antibiotics for patients allergic to penicillins and can also be used to treat infections caused by MRSA strains. Therefore, in recent years, an increase in the number of strains resistant to macrolides and lincosamides, in particular, the iMLS _B_ phenotype, has been observed [27,28].

Varshney et al. [29] reported that among strains derived from wounds, 5.6% are susceptible to all antibiotics and 40.7% are susceptible to all antibiotics, except for penicillin. We found similar percentages (6.0% and 35.0%, respectively). However, in the above-mentioned studies, in the case of strains isolated from blood, these percentages were lower compared to the results of our research (6.1% and 23.2% vs. 14.0% and 46.0%).

*S. aureus* pathogenicity correlates with bacteria’s capability to produce and secrete a variety of virulence factors that contribute to colonization, invasion, and damage of the host tissue and bacterial spread [30]. Many virulence factors and biofilm formation are under the control of the accessory gene regulator-quorum sensing system (*agr*-QS). Toxins or proteases secreted by *S. aureus* have an important role in the ability to cause disease, but may become redundant once a chronic infection is established. Because many virulence factors are targets for the immune system, their downregulation or loss of agr-qs prevents further recruitment of phagocytes so is crucial for bacterial survival and long-term persistence in host cells/tissue. Strains isolated from chronic infections may also rewire their metabolism to promote biofilm production or may survive in a metabolically inactive state forming small-colony variants [31,32,33].

In this study, among all the enterotoxin-encoding genes tested, *sei* and *seg* were the most frequent. A combination of those two genes was also found to be the most common. This is in accordance with the previous study [5,15,21,34,35,36] and related to the close location of both genes on the enterotoxin gene cluster (*egc*) [37]. A significantly higher percentage of *sei*, *seg* and *sea* was found in chronic wound strains than in blood strains (61.0%, 59.0% and 19.0% vs. 46.0%, 45.0% and 6.0%, respectively). A similar relationship was observed in another study [38], among strains isolated from wounds (56.3%, 56.3% and 43.8%) and blood (41.2%, 41.2% and 35.3%), whereas Pérez-Montarelo et al. [15] recorded a slightly lower percentage of strains causing SSTI harboring those genes compared to strains causing catheter-related bacteremia (67.7%, 68.2% and 30.8% vs. 72.8%, 75.6% and 31.4%, respectively). It is believed that *egc* superantigenes facilitate the colonization of mucosal surfaces and promote bacterial survival but negatively correlate with the severity of infections [39,40]. We also observed a higher rate of *sea* and *sed* genes among wound strains, which is in accordance with other data [41]. It is considered that *sea* and *sei* may be a biomarker to distinguish colonization and infection of a wound [38]. SEA and SED, with their increased ability to induce local inflammatory responses, in turn, may perform a function in perpetuating chronic non-healing wounds. Moreover, Merriman [11] demonstrated that superantigens can delay wound closure by altering cell proliferation and migration.

In our study, the distribution rate of genes other than *seg* and *sei* enterotoxin genes among blood strains was much lower (1.0–12.0%). These findings are inconsistent with those reported previously, where *sea* [15,22,39], *seb* [5,35,42], or *sec* [35] were also widely present. However, Jarraud et al. [43] suggested that *seg-sei* gene combination without any of the other SEs, TSST-1 and ETs may also be capable of causing diseases such as toxic shock syndrome or staphylococcal scalded skin syndrome.

Park et al. [36] found *eta* and *etb* genes in 2.6% and 100% SAB strains, respectively, in a two-year study period, but many studies [3,24,35,38,44] showed a higher distribution of *eta* compared to *etb*. This is in accordance with our results, where the *eta* gene was detected in 13.0–14.0% of the *S. aureus* strains, whereas only 0–1.0% strains harbored the *etb* gene. Li et al. [35] reported that both genes were more common in SAB isolates compared to SSTI isolates recovered from children. The frequency of occurrence of genes encoding exotoxins, however, varies among blood strains of *S. aureus* and may also be equal to or near zero [34,42,45,46].

The *luk-F/S-PV* gene is detected more often in SSTI isolates than in SAB isolates [15,26,32,45,46,47,48], which confirms our results. The incidence of *luk-F/S-PV* gene in this study, however, was not high (3.0%), unlike the results of other researchers, in which it reached about 80% [35,47]. These differences may result from testing only strains derived from chronic wounds compared to tests involving different types of SSTI. In the studies conducted on staphylococci isolated from patients with diabetic foot infections, Viquez-Molina et al. [49] detected the *luk-F/S-PV* gene in 6.9% of strains and, in another study [50], this gene was not found in any of the isolates.

TSST-1 produced by *S. aureus* has been associated with several acute diseases including toxic shock syndrome, but also with chronic diseases. Our data showed that the *tst* gene was carried by a relatively low (9.0%) percentage of blood strains, compared to the results of previous studies in which this percentage usually exceeded 10% [3,15,34,36,38,45,50,51]. Compared with SAB strains, the carriage rate for the *tst* gene in chronic wound strains was slightly higher (13.0%), although the difference was not significant. This is in agreement with the results obtained with strains isolated from DFU and other SSTI [15,20,26,38].

Compared to strains derived from chronic wounds, a high percentage (35.0%) of strains isolated from patients with SAB did not have any of the genes tested. A lower percentage (25.6% and 29.4%) of *S. aureus* strains obtained from blood without the virulence genes investigated was found by Becker et al. [34] and Demir et al. [38], respectively. In the present study a considerable variety of virulence profiles was observed among all strains, and only 12 of the 39 profiles coincided in both groups. The possession of single genes was observed in 18.0% and 15.0% of wound and blood strains, respectively. A similar result was reported in the study by Demir et al. [38].

We found that SEs genes were more frequent among MRSA than MSSA strains. Other studies also showed a significant association between methicillin resistance and the presence of the majority of SEs genes [5,21,36,42,52]. In our study, among the tested genes encoding enterotoxins tested, only *sea* was found in more MSSA strains than MRSA. Park et al. [36] found no *sea* gene in MRSA bacteremia isolates, whereas over 16% of MSSA strains carried this gene.

Our results support the results of other studies [23,36,50,53], in which a higher rate of *tst*-carrying isolates was detected among methicillin-susceptible isolates compared to that for MRSA isolates, which indicates a possible, inversely proportional relationship between drug resistance of the strains and their virulence in the occurrence of virulence genes.

To our knowledge, scientists most often analyze the difference in the prevalence of virulence genes in MSSA and MRSA strains, while the relationship between the presence of genes encoding toxins and drug susceptibility is less frequently assessed. No significant difference between the antibiotic resistance of non-enterotoxigenic strains and strains producing SEA, SEB, SEC, and SED from mastitic milk was reported by Suleiman et al. [54]. Corredor Arias et al. [55] drew similar conclusions from research results in which 22 superantigen genes were detected in strains isolated from different clinical samples. Choopani et al. [56] reported a relationship between SEG and SEI production and antibiotic resistance, as well as a correlation between *seb* gene presence and resistance to antibiotics in MRSA strains isolated from a clinical sample in a hospital in Tehran. In our study, an interesting fact was the detection of antibiotic resistance associated with the *sed* gene.

The association between the distribution of genes encoding virulence factors and the development of a range of staphylococcal infections is still unknown. Moreover, infections caused by multi-resistant *S. aureus* strains remain a challenge for clinicians due to the limited treatment options in patients with such infections. The present research investigated drug susceptibility and virulence patterns in *S. aureus* strains isolated from chronic wounds and blood. The limitation of the study was the inability to assess the expression of investigated virulence genes and perform molecular typing of the isolates, which would allow for a better interpretation of our findings. Often, antibiotic susceptibility pattern, virulence factors, and their combinations are associated with particular genetic backgrounds (clones) that carry specific pathogenicity islands or plasmids. Although those limitations exist, our results allow us to suggest that strains obtained from chronic wounds seem to be more often resistant to antibiotics and more virulent compared to strains isolated from blood. We observed a statistically significant difference in the incidence of *sea* and *sei* genes, and the percentage of strains resistant to amikacin, gentamicin and tobramycin. Further studies on the pathogenesis of *S. aureus*, including molecular typing, are required to elucidate the importance of various virulence factors in strains from patients with SAB and chronic non-healing wounds.

## 4. Materials and Methods

### 4.1. Bacterial Identification and Antibiotic Susceptibility

A total of 200 *S. aureus* strains (non-duplicated) isolated from 2015 to 2017 were analyzed in this study. One hundred strains were obtained from chronic wounds (diabetic foot ulcers and venous leg ulcers), the other 100 strains were isolated in the same period from the blood of patients hospitalized at dr. A. Jurasz University Hospital no. 1, L. Rydygier Collegium Medicum in Bydgoszcz, Nicolaus Copernicus University in Torun. The bacteria were identified using matrix-assisted laser desorption ionisation-time of flight mass spectrometry (MALDI-TOF MS) (Bruker, Bremen, Germany). An antimicrobial susceptibility test was carried out using the disk diffusion method recommended by the European Committee on Antimicrobial Susceptibility Testing (EUCAST) [57]. The following drugs were tested: benzylpenicillin (1 unit) (P), cefoxitin (30 µg) (FOX), ceftaroline (5 µg) (CPT), norfloxacin (10 µg) (NOR), amikacin (30 µg) (AMK), gentamicin (10 µg) (G), tobramycin (10 µg) (TOB), erythromycin (15 µg) (E), clindamycin (2 µg) (CC), tigecycline (15 µg) (TGC), linezolid (10 µg) (LZD), rifampicin (5 µg) (RF) and trimethoprim-sulfamethoxazole (25 µg) (SXT). Inducible macrolide, lincosamide and streptogramin B (iMLS_B_) resistance was demonstrated by the D-zone test. The minimum inhibitory concentration (MIC) of vancomycin and teicoplanin was determined by microdilutions in Mueller–Hinton broth. Isolates which showed resistance to non-susceptibility to at least one agent in three or more antimicrobial categories were classified as multidrug-resistant.

### 4.2. Toxin Genes Detection

Bacterial DNA was isolated with the Genomic Mini purification kit (A&A Biotechnology, Gdańsk, Poland) according to the manufacturer’s instruction. DNA extracts were used as a template for amplification. The following virulence genes were detected: the staphylococcal enterotoxin genes (*sea*, *seb*, *sec*, *sed*, *seg*, *sei*), the exfoliative toxin genes (*eta*, *etb*), the PVL genes (*luk-F/S-PV*), and the toxic shock syndrome toxin gene (*tst*). The presence of those genes was determined via PCR using oligonucleotide primers [43,58], listed in Table 6. Five monoplex PCR and two multiplex PCR assays, to detect simultaneously (1) *sec* and *tst*, (2) *seb*, *eta*, and *etb*, were designed, PCR amplifications were performed using the Mastercycler pro thermal cycler (Eppendorf, Stevenage, UK). The PCR mixture contained 1 µL DNA template, 0.8 µM (*seb*, *sei*, *eta*, *etb*) /1 µM (other genes) of primers (Laboratory of DNA Sequencing and Oligonucleotide Synthesis, IBB Polish Academy of Sciences), 1 x PCR buffer, 1.5 mM MgCl2, 200 µM dNTP mix (Promega, Madison, WI, USA) and 1 U (*sea*, *sed*, *seg*, *sei*, *luk-F/S-PV*) /2 U (other genes) of GoTaq polymerase (Promega, Madison, WI, USA). Amplification of genes was performed using the following conditions: initial denaturation at 94 °C for 3 min followed by 30 (*sea*, *seg*, *sei*, *luk-F/S-PV*)/ 35 (other genes) cycles of denaturation at 94 °C for 1 (*seb*, *sec*, *eta*, *etb*, *tst*, *luk-F/S-PV*) /2 min (other genes), annealing (Table 6) and extension at 68 °C (*seb*, *sec*, *eta*, *etb*, *tst*) /72 °C for 1 min. A 7 min elongation step at 72 °C was included at the end of the final cycle. *S. aureus* isolates harboring virulence genes obtained from the National Reference Centre for Staphylococci (Lyon, France) were used as the positive control strains for detecting virulence genes. Negative control was also included in each PCR run. The PCR products were separated by electrophoresis in a 1.5% agarose gel with SimplySafe (EURx, Gdańsk, Poland) and visualized in a UV transilluminator. GeneRulerTM100 bp DNA Ladder (Fermentas, Waltham, MA, USA) was used as a molecular weight marker.

### 4.3. Statistical Analysis

The summary statistics for categorical variables are presented as absolute and relative frequencies. The differences in categorical variables were tested using chi-square or Fisher’s exact test for independence. To study the dependence between the resistance to antibiotics and the presence of virulence genes and the sample origin (wound and blood), a generalized linear mixed effects model (GLMM) with a binomial distribution and logit link function has been applied. GLMM is a class of models that enable the modelling of clustered non-normal data of many kinds of response variables. The virulence genes and the origin of the *S. aureus* strain (blood or wound) have been included in the model as covariates and the antimicrobial resistance as the dependent variable. The antibiotics and the strains’ identifiers were included as random effects terms. First, the associations between the antimicrobial resistance and the presence of virulence genes and the *S. aureus* origin have been modelled for each covariate separately (univariable models). Subsequently, the model with multiple covariates was created (multivariable model). Model estimates are reported as odds ratios (ORs).

The results were considered statistically significant when the *p*-value was less than 0.05. The statistical analysis was performed with the use of the R software (package lme4) [59].

## Figures and Tables

**Table 1:** Comparison of the antimicrobial resistance between wound and blood *S. aureus* strains.

Antibiotic	Total No. of *S. aureus*, R (%)	Chronic Wound (*n* = 100), R (%)	Blood (*n* = 100), R (%)	*p* Value
Benzylpenicillin	171 (85.5)	86 (86.0)	85 (85.0)	1
Cefoxitin	44 (22.0)	21 (21.0)	23 (23.0)	0.865
Ceftaroline	0	0	0	1
Norfloxacin	54 (27.0)	31 (31.0)	23 (23.0)	0.265
Amikacin	43 (21.5)	31 (31.0)	12 (12.0)	0.002
Gentamicin	14 (7.0)	13 (13.0)	1 (1.0)	0.001
Tobramycin	46 (23.0)	32 (32.0)	14 (14.0)	0.004
Teicoplanin	0	0	0	1
Vancomycin	0	0	0	1
Erythromycin	59 (29.5)	30 (30.0)	29 (29.0)	1
Clindamycin	60 (30.0)	29 (29.0)	31 (31.0)	0.877
Tigecycline	0	0	0	1
Linezolid	0	0	0	1
Rifampicin	0	0	0	1
Trimethoprim-sulfamethoxazole	5 (2.5)	3 (3.0)	2 (2.0)	1

R—Resistance.

**Table 2:** Antimicrobial resistance patterns among wound and blood *S. aureus* strains.

Resistance Profile	Chronic Wound (*n* = 100) (%)		Blood (*n* = 100) (%)	
	MRSA	MSSA	MRSA	MSSA
Susceptible to all antibiotics	-	6 (6.0)	-	14 (14.0)
P	0	35 (35.0)	0	46 (46.0)
NOR	0	2 (2.0)	0	0
P, NOR	1 (1.0)	5 (5.0)	2 (2.0)	2 (2.0)
P, AMK	0	1 (1.0)	0	0
P, G	0	2 (2.0)	0	0
E, CC	0	3 (3.0)	0	1 (1.0)
AMK, TOB	0	2 (2.0)	0	0
P, AMK, TOB	1 (1.0)	5 (5.0)	1 (1.0)	3 (3.0)
P, NOR, AMK	0	1 (1.0)	0	0
P, NOR, SXT	0	0	1 (1.0)	0
P, E, CC	0	6 (6.0)	1 (1.0)	10 (10.0)
P, AMK, G, TOB	0	6 (6.0)	0	0
P, AMK, G, CC	0	0	1 (1.0)	0
P, NOR, E, CC	5 (5.0)	0	7 (7.0)	1 (1.0)
P, NOR, AMK, G, SXT	1 (1.0)	0	0	0
P, AMK, G, TOB, E	1 (1.0)	0	0	0
P, NOR, TOB, E, CC	3 (3.0)	0	3 (3.0)	0
P, NOR, AMK, G, TOB, SXT	0	1 (1.0)	0	0
P, NOR, AMK, TOB, E, CC	9 (9.0)	0	6 (6.0)	0
P, NOR, AMK, TOB, CC, SXT	0	0	1 (1.0)	0
NOR, AMK, G, TOB, E, CC	0	1 (1.0)	0	0
P, NOR, AMK, G, TOB, E, CC, SXT	0	1 (1.0)	0	0

P—Benzylpenicillin, NOR—Norfloxacin, AMK—Amikacin, TOB—Tobramycin, E—Erythromycin, CC—Clindamycin, FOX—Cefoxitin, G—Gentamicin, SXT—Trimethoprim-sulfamethoxazole.

**Table 3:** Frequencies of virulence genes among wound and blood strains and among MRSA and MSSA strains.

Virulence Genes	Total No. of *S. aureus* (%)	Chronic Wound (*n* = 100) (%)	Blood (*n* = 100) (%)	*p* Value	MRSA (*n* = 44) (%)	MSSA (*n* = 156) (%)	*p* Value
*sea*	25 (12.5)	19 (19.0)	6 (6.0)	0.009	2 (4.5)	23 (14.7)	0.071
*seb*	4 (2.0)	3 (3.0)	1 (1.0)	0.621	1 (2.3)	3 (1.9)	0.884
*sec*	15 (7.5)	8 (8.0)	7 (7.0)	1.000	5 (11.4)	10 (6.4)	0.271
*sed*	32 (16.0)	20 (20.0)	12 (12.0)	0.176	18 (40.9)	14 (9.0)	0.001
*seg*	104 (52.0)	59 (59.0)	45 (45.0)	0.066	34 (77.3)	70 (44.9)	0.001
*sei*	107 (53.5)	61 (61.0)	46 (46.0)	0.047	35 (79.5)	72 (46.2)	0.001
*tst*	22 (11.0)	13 (13.0)	9 (9.0)	0.499	4 (9.1)	18 (11.5)	0.647
*eta*	27 (13.5)	14 (14.0)	13 (13.0)	1,000	2 (4.5)	25 (16.0)	0.049
*etb*	1 (0.5)	0	1 (1.0)	1,000	0	1 (0.6)	0.594
*luk-F/S-PV*	4 (2.0)	3 (3.0)	1 (1.0)	0.621	1 (2.3)	3 (1.9)	0.884

**Table 4:** Distribution of virulence genes in chronic wound and blood *S. aureus* strains.

Gene Profile	Chronic Wound (%)	Blood (%)	Gene Profile	Chronic Wound (%)	Blood (%)
Lack of tested genes	19 (19.0)	35 (35.0)	*sed*, *seg*, *sei*	7 (7.0)	6 (6.0)
*sei*	6 (6)	1 (1.0)	*seg*, *sei*, *eta*	7 (7.0)	3 (3.0)
*seg*	5 (5.0)	2 (2.0)	*sec*, *seg*, *się*	4 (4.0)	2 (2.0)
*eta*	1 (1.0)	6 (6.0)	*seg*, *sei*, *tst*	1 (1.0)	4 (4.0)
*tst*	2 (2.0)	2 (2.0)	*sea*, *seg*, *sei*	2 (2.0)	1 (1.0)
*sea*	3 (3.0)	0	*sei*, *eta*, *luk-F/S-PV*	2 (2.0)	0
*sed*	0	3 (3.0)	*sea*, *eta*, *etb*	0	1 (1.0)
*seb*	1 (1.0)	0	*seg*, *sei*, *luk-F/S-PV*	0	1 (1.0)
*sec*	0	1 (1.0)	*sea*, *sed*, *sei*	1 (1.0)	0
*seg*, *sei*	15 (15.0)	21 (21.0)	*sea*, *seb*, *luk-F/S-PV*	1 (1.0)	0
*sei*, *eta*	3 (3.0)	0	*sec*, *sed*, *seg*, *sei*	1 (1.0)	2 (2.0)
*sed*, *seg*	3 (3.0)	0	*sea*, *seg*, *sei*, *tst*	3 (3.0)	0
*sea*, *eta*	0	2 (2.0)	*sea*, *sed*, *seg*, *sei*	2 (2.0)	0
*sei*, *tst*	0	2 (2.0)	*sea*, *seb*, *sed*, *seg*	1 (1.0)	0
*sea*, *seg*	1 (1.0)	1 (1.0)	*sed*, *seg*, *sei*, *tst*	1 (1.0)	0
*sea*, *sed*	0	1 (1.0)	*sec*, *seg*, *sei*, *eta*	0	1 (1.0)
*seb*, *sei*	0	1 (1.0)	*sec*, *seg*, *sei*, *tst*	0	1 (1.0)
*sea*, *sei*	1 (1.0)	0	*sea*, *sed*, *seg*, *sei*, *tst*	2 (2.0)	0
*seg*, *eta*	1 (1.0)	0	*sec*, *sed*, *seg*, *sei*, *tst*	2 (2.0)	0
*sea*, *tst*	1 (1.0)	0	*sea*, *sec*, *seg*, *sei*, *tst*	1 (1.0)	0

**Table 5:** Factors associated with the resistance to antibiotics.

Variable	Estimate	SE	Unadjusted OR (95%CI)	*p* Value	Estimate	SE	Adjusted OR (95%CI)	*p* Value
	Univariable models				Multivariable model			
chronic wound vs. blood	0.729	0.337	2.07 (1.07–4.01)	0.03	0.424	0.301	1.53 (0.85–2.75)	0.158
*sed*	2.565	0.405	13 (5.87–28.78)	<0.001	2.207	0.409	9.09 (4.08–20.27)	<0.001
*seg*	1.334	0.326	3.8 (2.01–7.19)	<0.001	0.854	0.417	2.35 (1.04–5.32)	0.04
*sei*	0.849	0.333	2.34 (1.22–4.48)	0.011	−0.13	0.406	0.88 (0.4–1.95)	0.749
*sea*	−0.495	0.514	0.61 (0.22–1.67)	0.336				

SE—Standard Error, OR (95% CI)—odds ratio with 95% confidence interval.

**Table 6:** Oligonucleotide primers used for amplification.

Target	Primer Sequence (5′→3′)	Product Size (bp)	Annealing Temp. (°C)	Reference
*sea*	GAAAAAAGTCTGAATTGCAGGGAACA	560	55	[58]
	CAAATAAATCGTAATTAACCGAAGGTTC			
*seb*	ATTCTATTAAGGACACTAAGTTAGGGA	404	57	[58]
	ATCCCGTTTCATAAGGCGAGT			
*sec*	GTAAAGTTACAGGTGGCAAAACTTG	297	56	[58]
	CATATCATACCAAAAAGTATTGCCGT			
*sed*	GAATTAAGTAGTACCGCGCTAAATAATATG	492	55	[58]
	GCTGTATTTTTCCTCCGAGAGT			
*seg*	AATTATGTGAATGCTCAACCCGATC	642	55	[43]
	AAACTTATATGGAACAAAAGGTACTAGTTC			
*sei*	CTCAAGGTGATATTGGTGTAGG	576	55	[43]
	AAAAAACTTACAGGCAGTCCATCTC			
*eta*	ACTGTAGGAGCTAGTGCATTTGT	190	57	[58]
	TGGATACTTTTGTCTATCTTTTTCATCAAC			
*etb*	CAGATAAAGAGCTTTATACACACATTAC	612	57	[58]
	AGTGAACTTATCTTTCTATTGAAAAACACTA			
*luk-F/S-PV*	ATCATTAGGTAAAATGTCTGGACATGATCCA	433	50	[58]
	GCATCAASTGTATTGGATAGCAAAAGC			
*tst*	TTCACTATTTGTAAAAGTGTCAGACCCACT	180	56	[58]
	TACTAATGAATTTTTTTATCGTAAGCCCTT			

## Data Availability

No new data were created or analyzed in this study. Data sharing is not applicable to this article.
